# Distinct Molecular Signatures of Amyloid-Beta and Tau in Alzheimer’s Disease Associated with Down Syndrome

**DOI:** 10.3390/ijms241411596

**Published:** 2023-07-18

**Authors:** Shojiro Ichimata, Ivan Martinez-Valbuena, Seojin Lee, Jun Li, Ali M. Karakani, Gabor G. Kovacs

**Affiliations:** 1 Tanz Centre for Research in Neurodegenerative Disease, University of Toronto, Toronto, ON M5T 2S8, Canada; shojiro.ichimata@mail.utoronto.ca (S.I.); ivan.martinez@utoronto.ca (I.M.-V.); lseojin.lee@mail.utoronto.ca (S.L.); junjl.li@utoronto.ca (J.L.); ali.karakani@utoronto.ca (A.M.K.); 2 Department of Laboratory Medicine and Pathobiology, University of Toronto, Toronto, ON M5S 1A1, Canada; 3 Department of Legal Medicine, Faculty of Medicine, University of Toyama, Toyama 930-8555, Japan; 4 Edmond J. Safra Program in Parkinson’s Disease, Rossy Program for PSP Research and the Morton and Gloria Shulman Movement Disorders Clinic, Toronto Western Hospital, Toronto, ON M5T 2S8, Canada; 5 Laboratory Medicine Program, Krembil Brain Institute, University Health Network, Toronto, ON M5G 2C4, Canada

**Keywords:** Alzheimer’s disease, amyloid-beta, chromosome 21, Down syndrome, p3 peptides, tau

## Abstract

Limited comparative data exist on the molecular spectrum of amyloid-beta (Aβ) and tau deposition in individuals with Down syndrome (DS) and sporadic Alzheimer’s disease (sAD). We assessed Aβ and tau deposition severity in the temporal lobe and cerebellum of ten DS and ten sAD cases. Immunohistochemistry was performed using antibodies against eight different Aβ epitopes (6F/3D, Aβ_38_, Aβ_39_, Aβ_40_, Aβ_42_, Aβ_43_, pyroglutamate Aβ at third glutamic acid (Aβ_Np3E_), phosphorylated- (p-)Aβ at 8th serine (Aβ_pSer8_)), and six different pathological tau epitopes (p-Ser202/Thr205, p-Thr231, p-Ser396, Alz50, MC1, GT38). Findings were evaluated semi-quantitatively and quantitatively using digital pathology. DS cases had significantly higher neocortical parenchymal deposition (Aβ_38_, Aβ_42_, and Aβ_pSer8_), and cerebellar parenchymal deposition (Aβ_40_, Aβ_42_, Aβ_Np3E_, and Aβ_pSer8_) than sAD cases. Furthermore, DS cases had a significantly larger mean plaque size (6F/3D, Aβ_42_, Aβ_Np3E_) in the temporal lobe, and significantly greater deposition of cerebral and cerebellar Aβ_42_ than sAD cases in the quantitative analysis. Western blotting corroborated these findings. Regarding tau pathology, DS cases had significantly more severe cerebral tau deposition than sAD cases, especially in the white matter (p-Ser202/Thr205, p-Thr231, Alz50, and MC1). Greater total tau deposition in the white matter (p-Ser202/Thr205, p-Thr231, and Alz50) of DS cases was confirmed by quantitative analysis. Our data suggest that the Aβ and tau molecular signatures in DS are distinct from those in sAD.

## 1. Introduction

Down syndrome (DS) is caused by triplication of chromosome 21, which harbors genes associated with Alzheimer’s disease (AD)-related proteins, including amyloid precursor protein (APP) and midbrain kinase/dual-specificity tyrosine phosphorylated and regulated kinase 1A (DYRK1A). As a result, most patients develop severe AD-like pathology early in life [1,2,3,4]. It has been suggested that the distribution and biochemical composition of amyloid-beta (Aβ) plaques and phosphorylated tau (p-tau)-immunoreactive neurofibrillary tangles (NFTs) in DS individuals with AD-type pathology are similar to those in familial and sporadic AD (sAD) [5,6]. Hence, DS is considered an ideal ‘model system’ for AD pathology [7]. However, we have recently reported some neuropathological differences between DS and sAD, including the distribution of NFTs and the morphology of Aβ plaques [8,9]. Moreover, Drummond et al. reported that DS cases had higher levels of post-translationally modified Aβ peptides, such as phosphorylated Aβ at the serine 8 residue (Aβ_pSer8_) and pyroglutamate Aβ at the third glutamic acid (Aβ_Np3E_) [10]. Likewise, Maxwell et al. found distinct and heterogeneous strains of Aβ in individuals with DS using principal component analysis [11]. Regarding tau, it has been reported that not only does the physiological phosphorylation of tau in DS differ significantly from that in normal controls [12], but also that the pathological processing of tau differs between DS and AD [13]. Furthermore, Condello et al. reported that the age-dependent kinetics of Aβ and tau are distinct in DS from those in AD [14]. Therefore, it is hypothesized that the molecular signatures of Aβ and tau in DS are different from those in sAD.

Cerebellar involvement represents the most severe form of Aβ deposition [15]. The morphology of cerebellar Aβ deposition is different from that in the cerebrum, as most Aβ deposits show a diffuse-type deposition and are not accompanied by tau deposition [8,16,17,18,19,20,21,22]. Recently, Miguel et al. reported that DS patients exhibited elevated cerebellar Aβ_42_ plaque load without any differences in Aβ plaque load labelled with a pan-Aβ antibody (clone 6E10; epitope lies within aa 4–9) compared to AD patients and healthy controls [23], suggesting that the molecular signatures of Aβ in the cerebellum may differ between DS and sAD.

In addition to the parenchyma, Aβ is deposited in cerebral blood vessels, forming cerebral amyloid angiopathy (CAA). Several studies reported that CAA pathology was more severe in DS than in AD [4,24,25]. Thus, the spectrum of deposited Aβ peptides may differ between DS and sAD. Although Reinert et al. examined these differences using immunohistochemical techniques with several antibodies against different Aβ peptides between DS and sAD cases, only three DS cases were included, and the differences were not shown in their study [26].

In recent years, amyloid-centric therapeutic strategies have been rapidly evolving [27,28]. Therefore, understanding the molecular signatures of pathological proteins deposited in each neurodegenerative disease is considered essential to further improve therapeutic efficacy. However, no study has evaluated the broad spectrum of Aβ and tau deposition and their interrelationships in human brain samples from DS and sAD. Here, we performed comprehensive immunohistochemistry using antibodies recognizing eight different epitopes of Aβ and six different pathological tau epitopes in a series of ten DS and ten sAD cases. In addition, biochemical experiments were performed to reinforce the immunohistochemical findings.

## 2. Results

### 2.1. Clinical Profiles and Demographics

The DS group consisted of four males and six females ranging from 42 to 66 years of age, whereas the sAD group consisted of six males and four females ranging from 75 to 94 years (Table 1).

There was no significant difference in the sex ratio between the two groups (*p* = 0.66). In contrast, the mean age was significantly younger in the DS group than in the sAD group (56.4 ± 7.1 vs. 80.8 ± 5.3; *p* < 0.01), and brain atrophy was significantly more severe in the DS group than in the sAD group (918 ± 139 vs. 1127 ± 142 g; *p* = 0.02). Nine out of ten patients had a high NIA-AA AD level in the DS group and eight out of ten in the sAD group. All cases showed CAA pathology in the temporal lobe (TL). In contrast, all DS cases and eight out of ten sAD cases exhibited cerebellar CAA pathology. Of note, white matter (WM) age-related tau astrogliopathy (ARTAG) was found only in the sAD group (sAD1, 4, 6, and 10) [29]. Additionally, one sAD case (AD10) showed additional neuropathological findings consistent with progressive supranuclear palsy (PSP) [30,31], and thus we excluded this case from the statistical analysis of tau pathology.

### 2.2. Aβ Pathology

**Figure 1:**
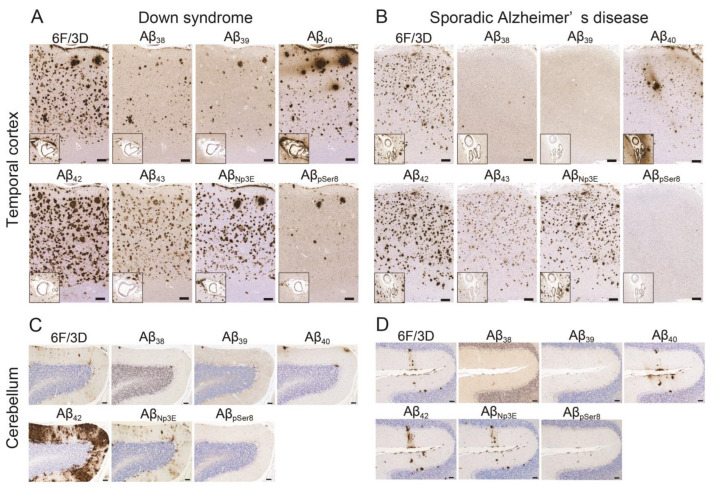
Representative microphotographs of Aβ pathology of Down syndrome (DS) and sporadic Alzheimer’s disease (sAD). (**A**,**B**) Temporal cortex. (**C**,**D**) Cerebellum. (**A**,**C**) DS case (Case No. DS2; see Table 1), (**B**,**D**) sAD case (Case No. sAD1; see Table 1). The inset shows a highly magnified view of the cerebral amyloid angiopathy (CAA) lesions in each immunostaining. (**A**,**B**) In the temporal cortex, Aβ plaque pathology is generally more severe in the DS case than in the sAD case. Note that plaques in the DS case are generally bigger than in the sAD case. (**C**,**D**) In the cerebellar molecular layer, the DS case show significantly severe Aβ_42_ deposition compared to the AD case. Note the stripe-like Aβ_42_ distribution in the molecular layer. No neuritic plaque formation is identified in the cerebellum. Immunostaining for Aβ_40_ showed immunoreactivity that spread from the plaque, especially around the bird-nest plaque (A), and CAA lesions to the surrounding area. Additionally, immunostaining for Aβ_38_, Aβ_39_, and Aβ_pSer8_ showed relatively higher background immunoreactivity in the parenchyma than the others. Scale bar = 250 μm (**A**,**B**), 100 μm (**C**,**D**).

**Figure 2:**
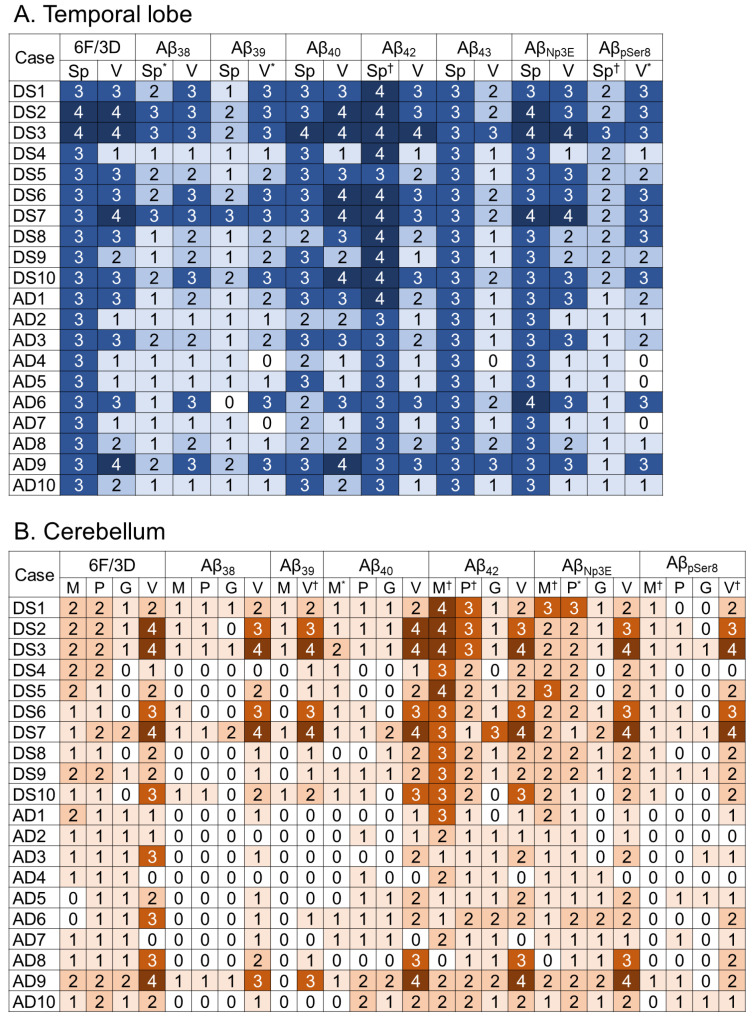
Semiquantitative Aβ pathology grading in the temporal lobe (**A**) and cerebellum (**B**). Darker blue and orange scale colour indicates greater severity as follows (for details see Section 4): 0 indicates no deposit; 1 minimal; 2 mild; 3 intermediate; 4 severe (**A**,**B**). Abbreviations: G, granular cell layer; M, molecular layer; P, Purkinje cell layer; Sp, senile plaque pathology; V, vascular pathology. * *p* < 0.05; † *p* < 0.01 (DS vs. AD, Mann–Whitney U test).

The representative immunohistochemical findings in the TL and cerebellum are shown in Figure 1, and the results of semi-quantitative grading are summarized in Figure 2.

In the TL, DS cases (Figure 1A) showed more severe plaque pathology than sAD cases (Figure 1B) in all Aβ-immunostainings investigated. The difference was statistically significant in immunohistochemistry for Aβ_38_, Aβ_42_, and Aβ_pSer8_ (Figure 2A; *p* = 0.04, < 0.01, and < 0.01, respectively). Additionally, the CAA pathology grading revealed generally more severe in DS cases than in sAD cases, but the difference reached statistical significance only in immunohistochemistry for Aβ_39_ and Aβ_pSer8_ (Figure 2A; *p* = 0.03 and 0.02, respectively).

In the cerebellum, as Purkinje cells (PCs) and granular cells (GCs) showed intracytoplasmic immunoreactivity for Aβ_39_, we only evaluated the pathological grading in the molecular layer (ML) and vessels for this immunostaining. Additionally, we excluded immunohistochemistry for Aβ_43_ from histopathological evaluation due to high background immunoreactivity. DS cases (Figure 1C) exhibited more severe plaque pathology than sAD cases (Figure 1D) in all types of Aβ-immunostaining investigated. The difference was statistically significant in immunohistochemistry for Aβ_40_, Aβ_42_, Aβ_Np3E_, and Aβ_pSer8_ (Figure 2B; *p* = 0.02, < 0.01, < 0.01, and < 0.01, respectively) in the ML, and for Aβ_42_ and Aβ_Np3E_ in the PC layer (PCL) (*p* < 0.01 and 0.04, respectively). In particular, DS cases showed significantly severe Aβ_42_ deposition (Figure 1C). In both groups, Aβ plaques in the ML exhibited diffuse-type morphology, and no neuritic plaque was identified. Regarding CAA pathology, DS cases showed generally more severe pathology than sAD cases, and the difference reached statistical significance in immunohistochemistry for Aβ_39_ and Aβ_pSer8_ (Figure 2B; *p* < 0.01 for both).

The quantitative analysis results are summarized in Table 2 (individual results are listed in Supplementary Table S1).

Given the amount of cerebral and cerebellar deposition and the non-specific background immunoreactivity, we quantitatively analyzed the deposition burden in specimens immunostained for 6F/3D, Aβ_42_, and Aβ_Np3E_. In the TL, the mean size of Aβ plaques was significantly larger in DS cases than in sAD cases. Additionally, the mean Aβ burden was higher in DS cases than in sAD cases in all three types of Aβ-immunostaining investigated, whereas the difference reached statistical significance only for Aβ_42_. These differences were also statistically significant in the cases with Braak NFT stage VI.

In the cerebellum, we excluded three sAD cases, because these cases did not show Aβ deposition immunoreactive for 6F/3D (sAD5 and 6) or Aβ_42_ and Aβ_Np3E_ (sAD8) in the ML. The mean Aβ burden was higher in DS cases than in sAD cases in all three types of Aβ-immunostaining investigated. Although the difference reached statistical significance only for Aβ_42_, DS cases tended to exhibit more severe Aβ_Np3E_ deposition than sAD cases.

**Figure 3:**
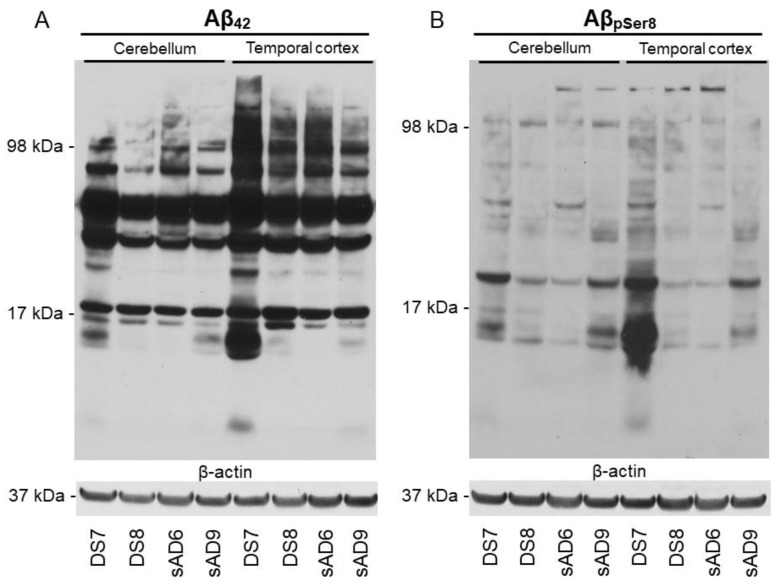
Results of Western blotting analysis. Biochemical evaluation of Aβ derived from Down Syndrome (DS) and sporadic Alzheimer’s disease (sAD) patients. Representative Aβ_42_ (**A**) and Aβ_pSer8_ (**B**) immunoblots showing the banding pattern of the PBS-soluble fraction from the cerebellum and temporal cortex extracts. Note that DS7 shows higher levels of Aβ_42_ and Aβ_pSer8_ than sAD cases both in the cerebellum and temporal cortex extracts.

To further confirm the histopathological findings, we performed Western blotting (Figure 3).

Consistent with the immunohistochemical results, one DS case (DS7) showed higher levels of Aβ_42_ (Figure 3A) and Aβ_pSer8_ (Figure 3B) than two sAD cases in the TL and cerebellum.

### 2.3. Tau Pathology

**Figure 4:**
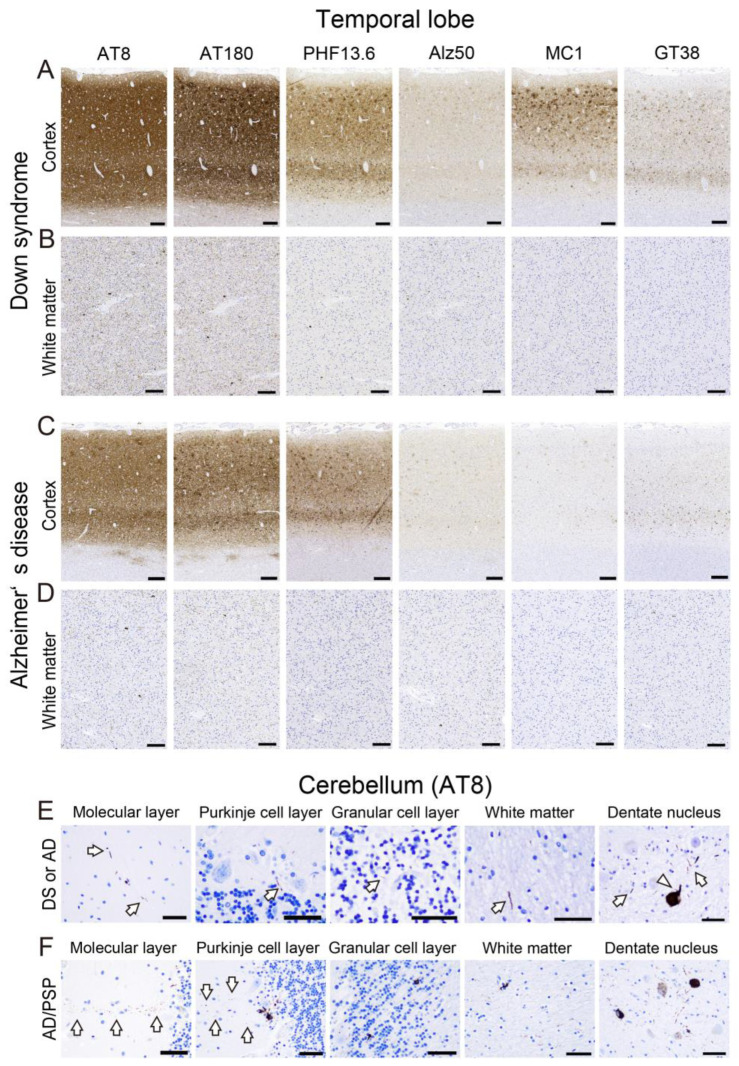
Representative microphotographs of tau pathology in the temporal lobe and cerebellum of DS and sAD. (**A**–**D**) Temporal cortex; (**E**,**F**) cerebellum (AT8). (**A**,**B**) DS case (Case No. DS1; see Table 1); (**C**,**D**) sAD case (Case No. sAD5; see Table 1). (**F**) sAD case with progressive supranuclear palsy (PSP) pathology (AD/PSP; Case No. sAD10; See Table 1). In the temporal lobe, cortical and white matter tau deposition is generally more severe in the DS case than in the sAD case. In contrast, in the cerebellum, only small thread-like (arrows) or dot-like lesions are observed in the molecular layer, Purkinje cell layer, granular cell layer, and WM in both DS and AD cases. In the dentate nucleus, threads and neuronal intracytoplasmic tau immunoreactivity are observed (arrowhead). (**F**) In the patient with PSP, band-like tau immunoreactivity perpendicular to the surface is observed in the ML (arrow). In addition, astrocytic tau deposition is observed in the Purkinje cell layer. Note the dot-shaped tau deposits in the molecular layer adjacent to the Purkinje cell layer lesion (arrow). Threads and coiled bodies are identified in the granular cell layer and white matter. The tau pathology in the dentate nucleus is also more severe in this case than in the other cases. Scale bar = 250 μm (**A**–**D**), 50 μm (**E**,**F**).

**Figure 5:**
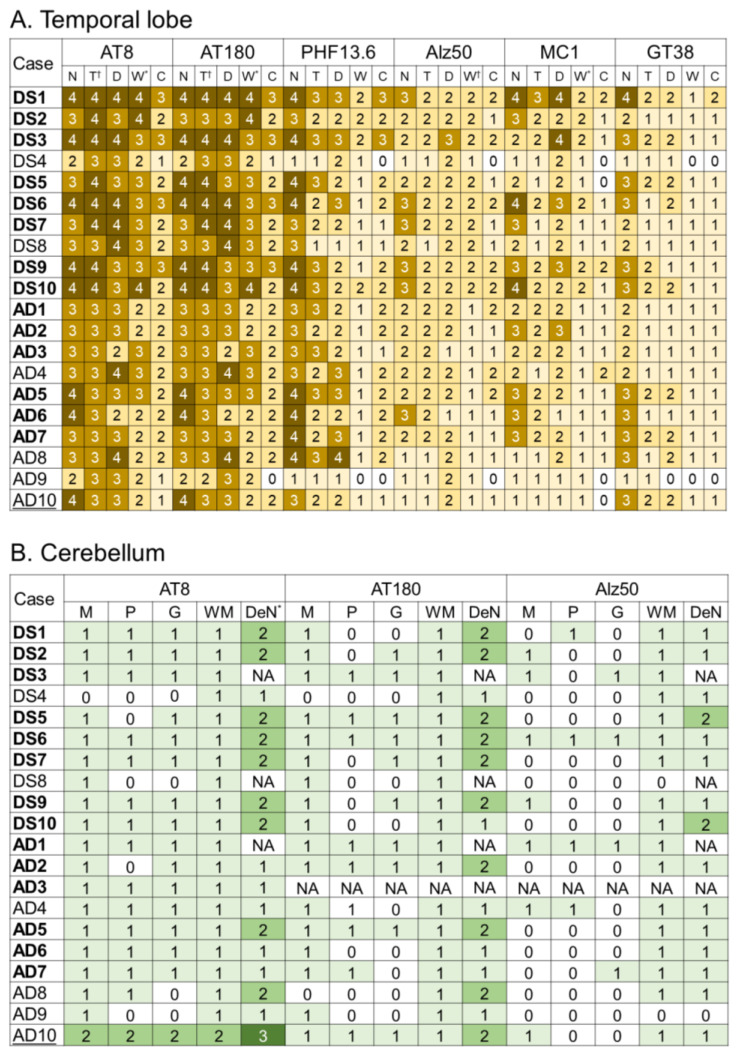
Semiquantitative tau pathology grading in the temporal lobe (**A**) and cerebellum (**B**). Darker yellow colour for the temporal lobe and green scale colour for the cerebellum indicates greater severity as follows: Grade 0, no tau pathology present; Grade 1, very few and scattered neuronal cytoplasmic tau immunoreactivity and neuropil threads; Grade 2, a mild number of neurofibrillary tangles and neuropil threads; Grade 3, a moderate number of neurofibrillary tangles and neuropil threads; and Grade 4, many densely packed neurofibrillary tangles and neuropil threads. (**A**,**B**). * *p* < 0.05; † *p* < 0.01 (DS vs. sAD, Mann–Whitney U test). Abbreviations: D, dystrophic neurite pathology; DeN, dentate nucleus; G, granular cell layer; M, molecular layer; N, neurofibrillary tangle pathology; NA, not available; P, Purkinje cell layer; T, neuropil threads pathology; W(M), white matter. Cases shown in **bold** have Braak neurofibrillary tangle pathology stage VI, and AD10 (underlined case) was excluded from the statistical analysis of tau pathology.

The representative immunohistochemical findings in the TL and cerebellum are shown in Figure 4, and the results of semi-quantitative grading are summarized in Figure 5.

In the TL, DS cases showed more severe tau pathology (Figure 4A,B) than sAD cases (Figure 4C,D) in all types of tau immunostaining investigated. In particular, DS cases showed significantly higher pathological grading of neuropil threads (NTs) in immunohistochemistry for AT8 and AT180 (Figure 5A; *p* < 0.01 for both), and WM pathological grading in immunohistochemistry for AT8, AT180, Alz50, and MC1 (Figure 5A; *p* = 0.01, 0.01, < 0.01, 0.03, respectively). Additionally, statistically significant differences were obtained in the pathological grading of dystrophic neurites (DNs) in immunohistochemistry for AT8 and AT180 when only cases exhibiting Braak NFT stage VI were examined (Figure 5A; *p* = 0.04 for both).

In the cerebellum, immunohistochemistry for AT8 revealed only a few immunoreactive lesions in the cortex and WM, and there was no apparent difference between DS and sAD cases (Figure 4E). In contrast, neuronal cytoplasmic tau immunoreactivity was observed in the dentate nucleus (DeN) (Figure 4E), and the DeN pathological grading was significantly more severe in DS cases than in sAD cases (Figure 5B; *p* = 0.04 in all cases; *p* = 0.02 in cases with Braak NFT stage VI). Considering these results, we additionally performed immunohistochemistry for AT180, which is superior to AT8 for detecting early lesions [32,33], and Alz50, which is a standard marker of tau conformational change [34]. However, there was no significant difference between the two groups (Figure 5B). As we expected, the case with AD/PSP pathology showed more prominent tau deposition than the others (Figure 5B). In particular, band-like and dot-shaped tau immunoreactivity, astrocytic tau immunoreactivity, and typical coiled bodies (CBs) in the GC layer (GCL) and WM were identified only in this case (Figure 4F).

The quantitative analysis results are summarized in Table 3 (all results are provided in Supplementary Table S2).

In the cortex, the mean total tau burden was higher in DS cases than in sAD cases in all types of tau immunostaining investigated. The difference in immunohistochemistry for AT180 was statistically significant only in cases with Braak NFT stage VI. In contrast, the mean total tau burden in the WM was higher in DS cases than in sAD cases in all types of tau immunostaining investigated, and the difference reached statistical significance in immunohistochemistry for AT8, AT180, and Alz50 in all cases and cases with Braak NFT stage VI. 

We further evaluated the seeding capacity of tau proteins extracted from the TL. However, we could not find any obvious difference between the two disease groups (Supplementary Figure S3).

### 2.4. Correlations between Various Aβ and Tau Deposition Burdens

Results of the correlation analysis between Aβ (6F/3D, Aβ_42_, and Aβ_Np3E_) and the neocortical tau (AT8, AT180, PHF13.6, Alz50, MC1, and GT38) quantitative deposition burdens in DS and AD cases are summarized in Table 4.

All types of Aβ burden values were positively correlated with all types of tau burden values, except for the combination of Aβ_Np3E_ and AT180 in sAD cases. Interestingly, sAD cases tended to have higher correlation coefficients for 6F/3D and Aβ_42_ burdens with most types of tau burdens than those in DS cases, with one pair of correlation coefficients reaching statistical significance (between Aβ_42_ and PHF13.6). In contrast, although none of these values reached statistical significance, DS cases tended to have higher correlation coefficients in the Aβ_Np3E_ burden with all types of tau burdens than in sAD cases.

## 3. Discussion

Here, we demonstrate that: (1) DS cases exhibited significantly higher neocortical parenchymal Aβ deposition (Aβ_38_, Aβ_42_, and Aβ_pSer8_) and cerebellar parenchymal Aβ deposition (Aβ_40_, Aβ_42_, Aβ_Np3E_, and Aβ_pSer8_) compared to sAD cases; (2) DS cases showed higher vascular Aβ deposition (Aβ_39_ and Aβ_pSer8_) than sAD cases in both regions; (3) DS cases had significantly more severe pathological tau deposition in the TL than sAD cases, particularly in the white matter (AT8, AT180, Alz50, and MC1); (4) DS cases displayed more severe tau deposition in the dentate nucleus than sAD cases; (5) the combinations of Aβ and tau burdens that demonstrated good correlations differed between DS and sAD cases.

There have been several neuropathological studies immunohistochemically examining the spectrum of deposited Aβ in DS [26,35,36,37,38,39]. However, only a small number of DS cases [26,39] or a few peptides [35,36,37] were examined in these studies. Although Saido et al. conducted a comprehensive analysis of the Aβ peptide spectrum in DS, they did not evaluate carboxyterminal truncated Aβ and p-Aβ peptides [39]. Moreover, there have been very few studies evaluating the spectrum of Aβ peptides deposited in the cerebellum using several anti-Aβ antibodies [23]. Furthermore, previous reports have examined the spectrum of either Aβ or tau in detail [13]. This is the first comprehensive immunohistochemical study examining differences in the spectrum of cerebral and cerebellar Aβ and tau pathologies between DS and sAD.

DS cases showed more severe deposition of major Aβ peptides (Aβ_40_ and Aβ_42_) as well as other Aβ peptides (Aβ_38_, Aβ_39_, Aβ_Np3E_, and Aβ_pSer8_) than sAD cases. Therefore, it is speculated that Aβ lesions in DS contain more diverse Aβ peptides than those in sAD, particularly minor peptides such as Aβ_38_, Aβ_39_ and Aβ_pSer8_. The emergence of heterogeneous Aβ strains in DS patients with advanced AD pathology may reflect this divergence [11]. Braun et al. reported that Aβ_42_ promotes the aggregation of shorter Aβ peptides, including Aβ_38_ [40]. Thus, the increased deposition of Aβ_42_ may be involved in the deposition of diverse Aβ peptides in DS. Interestingly, the size of Aβ plaques was significantly larger in DS cases than in sAD cases. One of the reasons for this difference may be the bird-nest plaque that is characteristic of DS [9]. Sepulveda-Falla et al. reported that the size of Aβ plaques in patients with presenilin-1 mutation E280A was larger than in early onset sAD cases [41]. Furthermore, the former patients had a significantly earlier age of onset and showed a higher amount of cortical Aβ_42_ deposition than the latter patients. Thus, several factors, including age, genetic abnormalities, the rate of disease progression, and the composition of Aβ peptides, may influence the size of Aβ plaques.

In the cerebellum, DS cases exhibited significantly higher deposition of Aβ_42_ and Aβ peptides with post-translational modifications (Aβ_Np3E_ and Aβ_pSer8_) than sAD cases. Deposition of these two post-translationally modified peptides corresponds to the most severe cerebral biochemical staging [42]. Therefore, the deposition of these Aβ peptides in the cerebellum also represents an advanced biochemical stage, and may have adverse effects on cerebellar functions. Additionally, most cerebellar Aβ_42_ deposits were not detected by the pan-Aβ antibody 6F/3D, supporting the results reported by Miguel et al. [23]. Therefore, it is presumed that cerebellar Aβ deposits are mainly composed of Aβ_17-42_ (so called p3 [43]). p3 is a major component of cerebral and cerebellar diffuse-type plaque [44,45], and is produced by α-secretase cleavage of APP [43,46]. Thus, p3 is typically considered non-amyloidogenic, although other recent studies have suggested that p3 is pathogenic [44,47,48]. In particular, Aβ_17-42_ contains 100% of the aggregation-prone ‘hot spots’ of Aβ_42_ [48]. Diffuse-type plaques are the earliest pathology, and can appear at 18 months of age in DS patients [49]. In addition, soluble Aβ_42_, including p3, is present even in fetal DS cases [45]. Thus, as with Aβ_42_ in the neocortex, increased p3 deposition may contribute to elevated deposition of other Aβ peptides in the cerebellum. Our observation of cerebellar Aβ pathology expands existing knowledge on cerebellar neurodevelopmental aspects of DS [50,51].

Wegiel et al. reported that overexpressed DYRK1A contributes to neurofibrillary degeneration in DS [2,3]. Consistent with their results, we found that DS cases exhibit significantly more severe tau pathology than sAD cases, despite having the same Braak stages of neurofibrillary degeneration, as shown in immunohistochemistry using antibodies that recognize p-tau. Notably, the differences were most pronounced in the WM. It has been reported that cerebral WM lesions are associated with cortical neurodegenerative pathology including AD [52,53]. Furthermore, Forrest et al. recently reported that neurodegeneration in the TL might be associated with pathological tau accumulation in the WM, including oligodendroglia [54]. Thus, it is presumed that DS, in which the formation of cortical AD-type lesions progresses more rapidly than in sAD, will show more severe WM lesions. Impaired myelination in DS may facilitate degeneration in the WM [55]. In our cohort, we did not detect ARTAG in the WM, which is frequently seen in sAD [29], in any DS cases, suggesting that the pathophysiology of ageing is also different between DS and sAD. These findings may be related to the altered physiological and pathological processing of tau in DS, which differs from controls and sAD [12,13]. Based on these findings and the observed differences in the immunostaining patterns of p-tau in DS compared to sAD (see AT180) at comparable Braak stages of neurofibrillary degeneration, therapy developers combining anti-Aβ and anti-tau therapies in DS should consider fine-tuning their strategies.

In both DS and sAD cases, tau pathology was minimal in the cerebellum, except in the DeN, corroborating the results of Jin et al. who analyzed tau seeding activity using two sensitive assays [56]. Interestingly, some tau pathologies, including band-like tau deposition, astrocytic intracytoplasmic tau deposition, and typical coiled-bodies, were identified only in the case of PSP pathology. Piao et al. reported similar findings in individuals with PSP and corticobasal degeneration, but not in cases with AD [57]. Therefore, the presence of these pathological findings may suggest the presence of tauopathy other than AD.

The severity of Aβ and tau burdens in the TL was positively correlated in most combinations of Aβ and tau profiles in the DS and AD group, supporting the existence of synergistic interactions between Aβ and tau in both diseases [58,59,60]. Interestingly, there were differences in the combinations of Aβ and tau burdens that showed good correlations between DS and sAD cases. Aβ pathology precedes the appearance of tau pathology in DS [61], while an opposite order has been recently proposed for sAD [7,62]. Our study supports the notion of distinct pathogenic interactions of Aβ and tau in distinct conditions with extracellular filamentous protein deposits [63]. As a limitation of our study, we were unable to assess longitudinal clinical scores and the impact of genetic alterations, such as *APOE*, on our findings.

In conclusion, we find that the molecular signatures of Aβ and tau in DS are distinct from those in sAD. Since dementia is now the leading cause of death in individuals with DS [64], establishing stratified and better-targeted treatment strategies for DS individuals is an urgent matter. Our study will inform therapy developers who aim to produce a more stratified therapeutic approach to conditions with combined Aβ and tau proteinopathy.

## 4. Materials and Methods

### 4.1. Case Selection

We examined ten DS cases and ten sAD cases, all with a neuropathological observation of frequent neuritic Aβ deposition (CERAD score C) [65] and cerebellar Aβ deposition (Thal Phase 5) [15], from the University Health Network Neurodegenerative Brain Collection (UHN-NBC). Selected case details are provided in Table 1. All brains had been obtained at autopsy through appropriate consenting procedures and with local ethical committee approval. This study was approved by the UHN Research Ethics Board (No. 20-5258) and the University of Toronto (No. 39459), and was performed per the ethical standards established in the 1964 Declaration of Helsinki, updated in 2008.

### 4.2. Immunohistochemistry

Formalin-fixed paraffin-embedded tissue sections from the TL (middle and superior temporal gyrus) and cerebellum (cut sagittally at the level of DN) were investigated immunohistochemically. Immunohistochemistry was performed using anti-Aβ antibodies including pan-Aβ (6F/3D; epitope lies within aa 10–15), antibodies detecting various peptides, such as Aβ_38_, Aβ_39_, Aβ_40_, Aβ_42_, Aβ_43_, Aβ_Np3E_ and Aβ_pSer8_, and anti-tau antibodies including p-Ser202/Thr205 (AT8), p-Thr231 (AT180), p-Ser396 (PHF13.6), Alz50, MC1, and GT38. Table 5 summarizes the immunohistochemistry methods used in this study.

### 4.3. Protein Extraction and Western Blotting

In two DS (DS7 and DS8) and two sAD cases (AD6 and AD9), frozen brain materials from the TL and cerebellum stored at −80 °C were available. Using a 4 mm brain tissue punch, a microdissection of the TL and cerebellum was performed, as previously described [66]. All the punches were stored in low protein binding tubes (Eppendorf, Hamburg, Germany) and immediately flash-frozen and stored at −80 °C. Then, 40–50 mg of frozen microdissected tissue was thawed on wet ice and then immediately homogenized in 500 μL of PBS spiked with protease (Roche, Basel, Switzerland) and phosphatase inhibitors (Thermo Scientific, Waltham, MA, USA) in a gentle-MACS Octo Dissociator (Miltenyi BioTec, Auburn, CA, USA). The homogenate was transferred to a 1.5-mL low protein binding tube (Eppendorf) and centrifuged at 10,000× *g* for 10 min at 4 °C, as previously described [66,67]. The supernatant was collected and aliquoted in 0.5-mL low protein binding tubes to avoid excessive freeze–thaw cycles. A bicinchoninic acid protein assay (Thermo Scientific) was performed to determine the total protein concentration of all samples. Gel electrophoresis was performed using 12% and 4–12% Bolt Bis-Tris Plus gels (Thermo Scientific). Proteins were transferred to 0.45-μm nitrocellulose membranes for 60 min at 30 V. The membranes were blocked for 60 min at room temperature in blocking buffer (5% [*w*/*v*] skimmed milk in 1× TBST (TBS and 0.05% [*v*/*v*] Tween-20)), and then incubated overnight at 4 °C with primary antibodies directed against Aβ_42_ (1:1000 dilution, ref: 14974S, Cell Signaling, Danvers, MA, USA) or against Aβ_pSer8_ (1:1000 dilution, ref: MABN878, St. Louis, MO, USA), diluted in the blocking buffer. The membranes were washed three times with TBST and then incubated for 60 min at room temperature with horseradish peroxidase-conjugated secondary antibodies (1:3000 dilution, ref: 172-1011 and 170-6515, Bio-Rad, Hercules, CA, USA) in the blocking buffer. Following another three washes with TBST, immunoblots were developed using Western Lightning-enhanced chemiluminescence Pro (PerkinElmer, Waltham, MA, USA), and imaged using X-ray films.

### 4.4. Pathological Assessment and Semiquantitative Grading System of Aβ and Tau Pathology

Following tau immunostaining (AT8), all cases were staged according to Braak’s NFT stage of burden [68]. Based on the results, the level of AD neuropathological change was categorized into four groups (not, low, intermediate, and high) using the National Institute on Aging-Alzheimer’s Association (NIA-AA) guidelines [69]. Additionally, we evaluated the type of cerebral amyloid angiopathy [70].

In addition to the staging systems described above, we semi-quantitatively assessed the severity of immunohistochemical findings of Aβ and tau. The severity of Aβ pathology in the brain parenchyma (as amyloid plaques) and cerebellar cortex, including the ML, PCL, GCL, and cerebral vessels (as CAA), was evaluated using a five-point scoring system [5,8], as follows: Plaque Grade 0, no Aβ plaques in parenchyma/layer; Grade 1, a few Aβ plaques in parenchyma/layer occupying each low-power (×10 microscope objective) field; Grade 2, a moderate number of Aβ plaques in parenchyma/layer occupying each low-power (×10 microscope objective) field; Grade 3, many dispersed Aβ plaques in parenchyma/layer occupying each low-power (×10 microscope objective) field; and Grade 4, very many densely packed Aβ plaques in parenchyma/layer occupying each low-power (×10 microscope objective) field. The CAA severity was evaluated using the following scoring system: Grade 0, no CAA in blood vessel walls in leptomeninges or brain parenchyma; Grade 1, occasional blood vessels with CAA in leptomeninges and/or within brain parenchyma, usually not occupying the full thickness of the wall; Grade 2, a moderate number of blood vessels with CAA in leptomeninges or brain parenchyma in leptomeninges or within brain parenchyma, some occupying the full thickness of the wall; Grade 3, many blood vessels with CAA in leptomeninges or brain parenchyma, most occupying the full thickness of the wall; and Grade 4, most or all blood vessels with severe CAA in leptomeninges or within brain parenchyma, occupying the full thickness of the wall.

In the TL, tau pathology severity was separately graded in the NFTs, NTs, DNs, and WM depositions using a five-point scoring system [5,8], as follows: Grade 0, no tau pathology present; Grade 1, very few and scattered neuronal cytoplasmic tau immunoreactivity and neuropil threads in each low-power (×10 microscope objective) field; Grade 2, a mild number of neurofibrillary tangles and neuropil threads in each low-power field; Grade 3, a moderate number of neurofibrillary tangles and neuropil threads in each low-power field; and Grade 4, many densely packed neurofibrillary tangles and neuropil threads in each low-power field. Using the same semiquantitative grading system, we also evaluated the pathological grading of CB formation in the WM.

### 4.5. Image Analysis

In the TL, we assessed the grey matter, wherein six layers of the cortex and the grey–white matter boundary are distinct, and show adequate immunoreactivity. In the WM, we selected areas in which only thread-like and oligodendroglial pathology were present. In each immunohistochemistry panel for tau and Aβ, the deposition burden was measured in the same region using serial sections. Representative photographs of the analysis regions are shown in Supplementary Figures S1 and S2. All immunostained sections were scanned at a magnification of 40× with a TissueScope LE120 slide scanner (Huron Digital Pathology, Ontario, ON, Canada), and the area containing the region of interest was cropped using the rectangular tool in the Huron Viewer under a magnification of 2.63× for the cortex analysis and of 12.5× for the WM analysis.

In the cerebellum, since Aβ deposition is predominantly present in the ML, we quantitatively evaluated the Aβ deposition burden only in this layer. Unlike the neocortex, Aβ deposition was patchy and unevenly distributed, especially in sAD cases. Therefore, we photographed five locations with the most severe Aβ deposition using a bright field microscope (area per image: 1.7 × 1.2 mm), and calculated the mean Aβ burden value. Microphotographs were acquired using a Nikon Eclipse Ci microscope, equipped with a DS-Fi3 microscope camera and NIS-Elements imaging software (Version 1.10.00; Nikon Instruments Inc., Tokyo, Japan).

All images were saved as TIFF files and then imported to Photoshop (Version 22.5.8; Adobe Inc., San Jose, CA, USA) separately. The regions of interest were digitally dissected and saved as new images. The new images were pre-processed by adjusting the contrast to improve the recognition of small deposits and reduce the background noise of non-specific immunoreactivity. Then, the Aβ and tau burdens in each image were quantified using Image J/Fiji (Image J Version 1.53r) [71], as described previously [12,72].

### 4.6. Tau Seeding Assay

The in vitro seeding assay was performed as previously described [73,74]. Briefly, the Tau RD P301S FRET Biosensor (ATCC CRL-3275) cells were cultured at 37 °C, 5% CO_2_ in DMEM, 10% vol/vol FBS, 0.5% vol/vol penicillin–streptomycin. Cells were plated on Ibidi clear-bottom 96-well plates at a density of 40,000 cells per well. Brain extracts (12 mg of total protein per well) were then incubated with Lipofectamine 2000 (Invitrogen, final concentration 1% vol/vol) in opti-MEM for 10 min at room temperature before being added to the cells. Each brain region was tested in duplicate. After 48 h, cells were fixed and imaged in 3 × 3 fields at 20× magnification using a Nikon ECLIPSE Ti2 confocal microscope. The total number of cells (DAPI) in the monolayer and tau aggregates (FITC) were quantified using the object colocalization IF module of HALO software (version 3.5, Indica Labs, Albuquerque, New Mexico) to calculate the number of seeded aggregates per cell (FITC/DAPI).

### 4.7. Statistical Analysis

Data were analyzed using IBM SPSS statistics version 26 (SPSS Inc., Chicago, IL, USA), and the significance level was set at 0.05. Fisher’s exact test was used for categorical variables (sex). Continuous variables (age at death and brain weight) and ordinal variables (semiquantitative pathological grading and quantitative deposition burden) were compared using a Mann–Whitney U test. Additionally, a Spearman’s rank correlation coefficient test was applied to examine the relationship between the quantitative Aβ and tau burden.

## Figures and Tables

**Table 1:** Demographic data of the individuals with Down syndrome (DS) and sporadic Alzheimer’s disease (sAD).

Case	Age **	Gender	BW (g) *	Braak NFT Stage	Thal Phase	CERAD Score	NIA-AA AD Level	CAA Type
DS1	42	F	900	6	5	C	High	2
DS2	49	M	1100	6	5	C	High	2
DS3	51	F	750	6	5	C	High	1
DS4	54	M	1130	3	5	C	Int	2
DS5	57	F	756	6	5	C	High	2
DS6	58	M	1100	6	5	C	High	1
DS7	61	M	905	6	5	C	High	1
DS8	61	F	900	5	5	C	High	2
DS9	65	F	880	6	5	C	High	1
DS10	66	F	760	6	5	C	High	1
sAD1	77	M	1200	6	5	C	High	2
sAD2	83	F	1000	6	5	C	High	2
sAD3	78	M	1220	6	5	C	High	2
sAD4	77	F	1200	5	5	C	High	2
sAD5	79	M	1200	6	5	C	High	2
sAD6	78	M	N/A	6	5	C	High	2
sAD7	94	F	830	6	5	C	High	2
sAD8	82	F	N/A	5	5	C	High	2
sAD9	75	M	1240	4	5	C	Int	1
sAD10	85	M	N/A	4	5	C	Int	2

Abbreviations: BW, brain weight; CAA, cerebral amyloid angiopathy; CBM, cerebellum; F, female; M, male; N/A, not available; NFT, neurofibrillary tangle; NIA-AA, the National Institute on Aging-Alzheimer’s Association; TL, temporal lobe. * *p* < 0.05; ** *p* < 0.01 comparison between DS and AD cases (Mann–Whitney U test).

**Table 2:** Summary of the quantitative Aβ plaque size and burden analysis results.

Plaque	Antibodies	DS	sAD	*p*-Value *
Cerebral Aβ burden in all cases (N = 10 cases, each)				
Size (range)	6F/3D	59.4 ± 13.3 (42.0–77.3)	36.8 ± 12.7 (15.6–55.0)	**<0.01**
	Aβ_42_	83.2 ± 21.5 (38.4–111.4)	50.6 ± 17.0 (28.2–75.5)	**<0.01**
	Aβ_Np3E_	97.5 ± 38.4 (46.1–173.2)	57.1 ± 28.3 (25.5–124.8)	**0.02**
Load (range)	6F/3D	7.6 ± 4.6 (1.8–16.2)	4.7 ± 1.8 (2.1–8.2)	0.22
	Aβ_42_	18.3 ± 6.7 (10.2–28.0)	8.3 ± 2.6 (4.5–12.0)	**<0.01**
	Aβ_Np3E_	8.9 ± 4.8 (1.8–18.2)	6.1 ± 2.0 (3.7–10.1)	0.19
Cerebral Aβ burden in cases with Braak NFT stage VI (N = 8 and 6 cases, respectively)				
Size (range)	6F/3D	60.0 ± 13.8 (42.0–77.3)	38.7 ± 10.2 (25.0–52.1)	**0.04**
	Aβ_42_	78.1 ± 21.1 (38.4–111.4)	49.3 ± 17.0 (28.2–75.5)	**0.03**
	Aβ_Np3E_	105.7 ± 37.7 (56.8–173.2)	52.3 ± 19.5 (25.5–84.0)	**<0.01**
Load (range)	6F/3D	9.0 ± 4.0 (4.4–16.2)	5.8 ± 1.3 (4.3–8.2)	0.18
	Aβ_42_	20.2 ± 6.2 (10.7–28.0)	9.0 ± 2.6 (5.0–12.0)	**<0.01**
	Aβ_Np3E_	10.3 ± 4.2 (5.2–18.2)	7.0 ± 2.1 (3.7–10.1)	0.18
Cerebellar molecular layer Aβ burden (N = 10 and 7 cases, respectively)				
Load (range)	6F/3D	2.0 ± 1.6 (0.2–5.1)	0.9 ± 0.8 (0.1–3.4)	0.16
	Aβ_42_	35.3 ± 11.8 (20.7–55.6)	8.7 ± 8.4 (1.6–28.4)	**<0.01**
	Aβ_Np3E_	4.9 ± 2.2 (2.0–8.2)	2.2 ± 1.3 (0.1–5.5)	0.06

**Boldface** signifies values that are significant at *p* < 0.05. * Comparison between the DS and sAD groups using Mann–Whitney U test.

**Table 3:** Summary of the quantitative tau burden analysis results.

Antibody	DS-Cx	sAD-Cx	DS-WM	sAD-WM	*p*-Value (Cx/WM) *
Mean total deposition burden in all cases (range) [N = 10 and 9 cases, respectively]					
AT8	56.9 ± 19.1 (16.9–77.0)	46.2 ± 13.9 (15.3–65.2)	6.0 ± 2.8 (1.0–10.9)	2.4 ± 1.2 (0.5–4.6)	0.18/<**0.01**
AT180	53.4 ± 18.9 (12.4–73.9)	39.5 ± 13.0 (13.7–56.2)	5.8 ± 2.6 (0.9–10.1)	2.2 ± 1.1 (0.6–4.0)	0.07/<**0.01**
PHF13.6	21.7 ± 16.0 (0.9–45.6)	18.9 ± 9.9 (0.2–38.7)	NA **	NA **	0.60/NE
Alz50	5.7 ± 3.6 (1.3–12.6)	3.9 ± 2.2 (1.0–7.3)	1.2 ± 0.6 (0.3–2.6)	0.6 ± 0.3 (0.1–1.2)	0.40/**0.02**
MC1	6.3 ± 8.6 (0.4–30.6)	2.2 ± 1.9 (0.2–6.3)	0.5 ± 0.4 (0.2–1.6)	0.4 ± 0.2 (0.2–0.8)	0.24/0.28
GT38	3.1 ± 3.1 (0.0–8.6)	0.9 ± 0.8 (0.0–2.4)	0.2 ± 0.2 (0.0–0.9)	0.2 ± 0.2 (0.0–0.7)	0.32/0.97
Mean total deposition burden in Braak NFT stage VI cases (range) [N = 8, 6 cases, respectively]					
AT8	64.2 ± 12.5 (44.0–77.0)	50.9 ± 10.4 (32.7–65.2)	6.8 ± 2.4 (3.6–10.9)	2.8 ± 1.1 (1.0–4.6)	0.11/<**0.01**
AT180	61.1 ± 11.0 (38.3–73.9)	43.5 ± 11.2 (22.7–56.2)	6.6 ± 2.1 (4.1–10.1)	2.6 ± 1.0 (0.9–4.0)	**0.01**/<**0.01**
PHF13.6	26.8 ± 13.6 (2.9–45.6)	22.8 ± 7.9 (12.5–38.7)	NA **	NA **	0.49/NE
Alz50	6.7 ± 3.4 (2.8–12.6)	5.1 ± 1.5 (3.3–7.3)	1.3 ± 0.6 (0.9–2.6)	0.7 ± 0.3 (0.4–1.2)	0.76/<**0.01**
MC1	7.7 ± 9.0 (1.0–30.6)	3.1 ± 1.7 (1.4–6.3)	0.6 ± 0.4 (0.2–1.6)	0.4 ± 0.2 (0.2–0.8)	0.41/0.57
GT38	3.9 ± 3.0 (0.1–8.6)	1.1 ± 0.9 (0.1–2.4)	0.1 ± 0.1 (0.0–0.3)	0.2 ± 0.2 (0.0–0.7)	0.18/1.00

**Boldface** signifies values that are significant at *p* < 0.05. Abbreviations: Cx, cortex; NA, not available; NFT, neurofibrillary tangle; WM, white matter. * Comparison between the DS and sAD groups using Mann–Whitney U test. ** Mean values could not be measured due to intense nonspecific background immunoreactivity.

**Table 4:** Summary of correlation analysis between various types of Aβ and tau deposition burdens in the DS group and the sAD group.

	6F/3D (r/*p*-Value)		Aβ_42_ (r/*p*-Value)		Aβ_Np3E_ (r/*p*-Value)	
	DS	sAD	DS	sAD	DS	sAD
AT8	0.42/0.23	0.50/0.17	0.50/0.14	0.65/0.06	0.42/0.23	0.02/0.97
AT180	0.36/0.31	0.47/0.21	0.38/0.28	0.63/0.07	0.33/0.35	0.00/1.00
PHF13.6	0.33/0.35	0.50/0.17	0.43/0.21	0.68/**0.04**	0.33/0.35	0.27/0.49
Alz50	0.30/0.41	0.62/0.08	0.37/0.29	0.12/0.77	0.35/0.33	0.27/0.49
MC1	0.37/0.29	0.55/0.13	0.49/0.15	0.20/0.61	0.43/0.21	0.22/0.72
GT38	0.19/0.60	0.25/0.52	0.24/0.51	0.30/0.43	0.16/0.65	0.02/0.97

For each combination of Aβ and tau deposition burdens in the comparison of DS and sAD groups, the higher correlation coefficient is indicated by underlined values, with significant values at *p* < 0.05 highlighted in **boldface** (according to a Spearman’s rank correlation coefficient test).

**Table 5:** Summary of antibodies used in this study.

Antibody	Source	Clone	Dilution	1st Antigen Retrieval	2nd Antigen Retrieval
Aβ_aa8–17_	Dako (Santa Clara, CA, USA)	6F/3D	1:50	80% FA 60 min	None
Aβ_38_	Synaptic Systems (Göttingen, Germany)	Polyclonal	1:1000	Heat	88% FA 3 min
Aβ_39_	Cell Signaling (Danvers, MA, USA)	D5Y9L	1:500	Heat	88% FA 3 min
Aβ_40_	BioLegend (San Diego, CA, USA)	QA18A67	1:2000	70% FA 10 min	None
Aβ_42_	BioLegend	1-11-13	1:500	70% FA 10 min	None
Aβ_43_	IBL (Fujioka, Japan)	Polyclonal	1:100	88% FA 5 min	None
Aβ_Np3E_	BioLegend	337.48	1:800	Heat	88% FA 3 min
Aβ_pSer8_	Sigma-Aldrich (St. Louis, MO, USA)	1E4E11	1:200	Heat	88% FA 3 min
p-tau (Ser202, Thr205)	Thermo Fischer (Waltham, MA, USA)	AT8	1:1000	Heat	None
p-tau (Thr 231)	Thermo Fischer	AT180	1:1000	Heat	None
p-tau (Ser396)	Thermo Fischer	PHF13.6	1:500	Heat	None
Anti-tau (Alz50)	Gifted *	Alz50	1:100	Heat	None
Anti-tau (MC1)	Gifted *	MC1	1:500	Heat	None
Anti-tau AD antibody	Abcam (Cambridge, UK)	GT38	1:1000	Heat *	None

Abbreviations: aa, amino acid; Aβ, amyloid-beta; AD, Alzheimer’s disease; FA, formic acid. Immunostaining was performed using the Dako Autostainer Link 48 and EnVision FLEX+ Visualization System, according to the manufacturer’s instructions. Subsequently, all sections were counterstained with haematoxylin. Heat-mediated antigen retrieval was performed using Dako PT Link with low pH solution. * Gift from Dr. Peter Davis.

## Data Availability

The datasets used and analyzed during the current study are available from the corresponding authors upon request.

## Supplementary Materials

The following supporting information can be downloaded at: https://www.mdpi.com/article/10.3390/ijms241411596/s1.

## Abbreviations

Aβ, amyloid-beta; Aβ_Np3E_, Aβ with a pyroglutamyl post translational modification at the third glutamate position; Aβ_pSer8_, Aβ peptide phosphorylated at serine 8 residue; AD, Alzheimer’s disease; ARTAG, age-related tau astrogliopathy; CAA, cerebral amyloid angiopathy; DN, dystrophic neurite; DS, Down syndrome; GCL, granular cell layer; ML, molecular layer; NFT, neurofibrillary tangle; NT, neuropil thread; p-, phosphorylated; PCL, Purkinje cell layer; PSP, progressive supranuclear palsy; sAD, sporadic Alzheimer’s disease; TL, temporal lobe; WM, white matter.
