# Supplementary material for: Distinct Molecular Signatures of Amyloid-Beta and Tau in Alzheimer’s Disease Associated with Down Syndrome
Source: Int J Mol Sci. 2023 Jul 18;24(14):11596. doi: 10.3390/ijms241411596 (PMC10380583; doi:10.3390/ijms241411596)
Supplement: Supplementary file 1 [file ijms-24-11596-s001.zip › ijms-2485386-supplementary.pdf]

Online Supplementary files

Distinct Molecular Signatures of Amyloid-beta and Tau in Alzheimer's Disease Associated with Down Syndrome

Shojiro Ichimata,[1,2,3] Ivan Martinez-Valbuena,[1] Seojin Lee,[1] Jun Li,[1] Ali M. Karakani,[1] Gabor G. Kovacs[1,2,4,5]

[1] Tanz Centre for Research in Neurodegenerative Disease, University of Toronto, Toronto, ON M5T 2S8, Canada;

[2] Department of Laboratory Medicine and Pathobiology, University of Toronto, Toronto, ON M5S 1A1, Canada

[3] Department of Legal Medicine, Faculty of Medicine, University of Toyama, Toyama 930-8555, Japan

[4] Edmond J. Safra Program in Parkinson’s Disease, Rossy Program for PSP Research and the Morton and Gloria Shulman Movement Disorders Clinic, Toronto Western Hospital, Toronto, ON M5T 2S8, Canada

[5] Laboratory Medicine Program, Krembil Brain Institute, University Health Network, Toronto, ON M5G 2C4, Canada

Supplementary Figure S1. Representative microphotographs of the quantitative analysis areas in the neocortex and cerebellar molecular layer

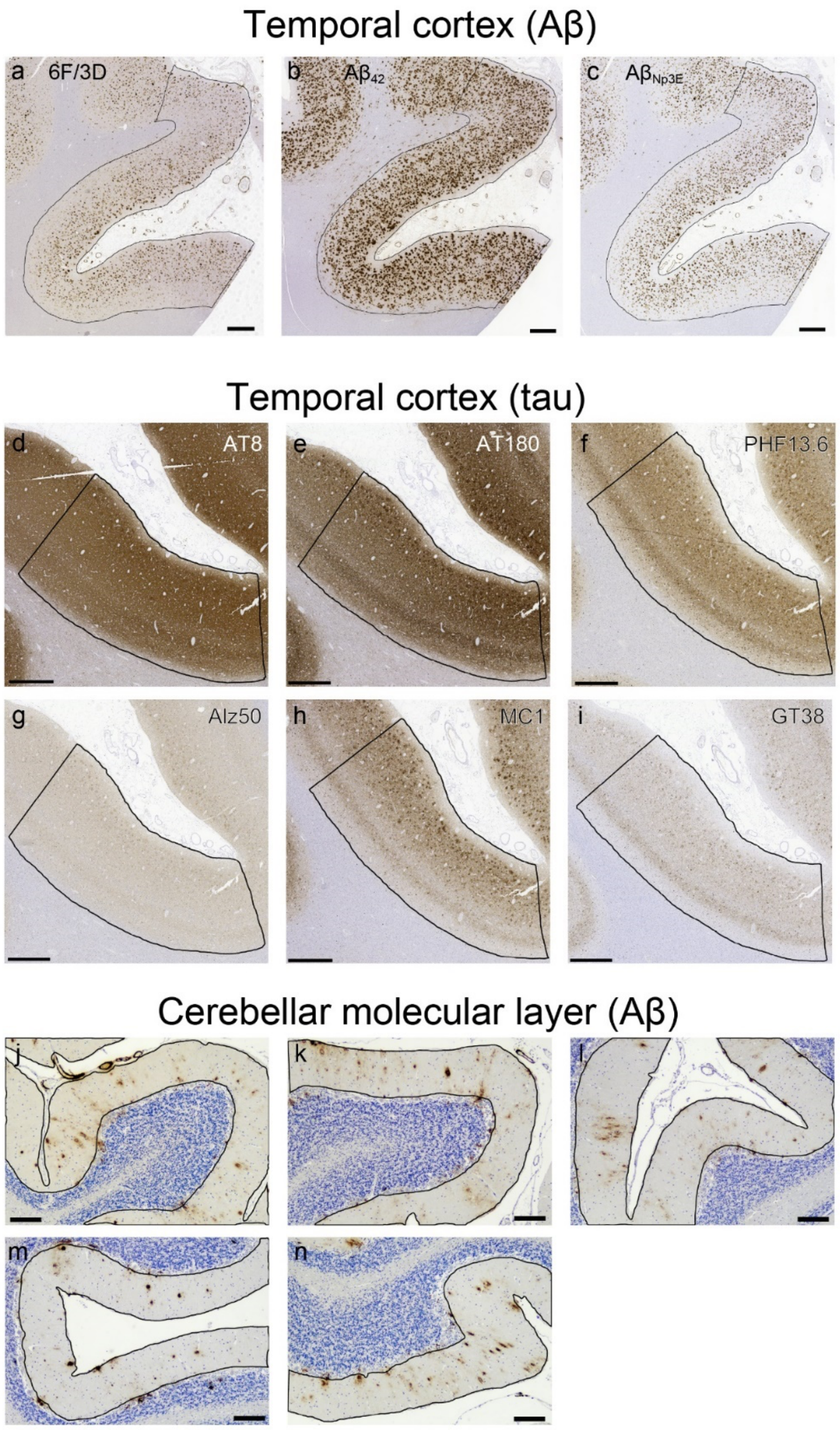

(a–n) DS1. (j–n) Immunohistochemistry for pan-Aβ (6F/3D). In each image, the area surrounded by the black line is the region of interest. In the quantitative analysis of Aβ in the temporal cortex, we evaluated the cortex including the sulcal depth and gyral crests to evaluate all types of Aβ plaques. Scale bar = 1 mm (a–i), 200 μm (j–n)

Supplementary Figure S2. Representative microphotographs of the quantitative analysis areas in the white matter.

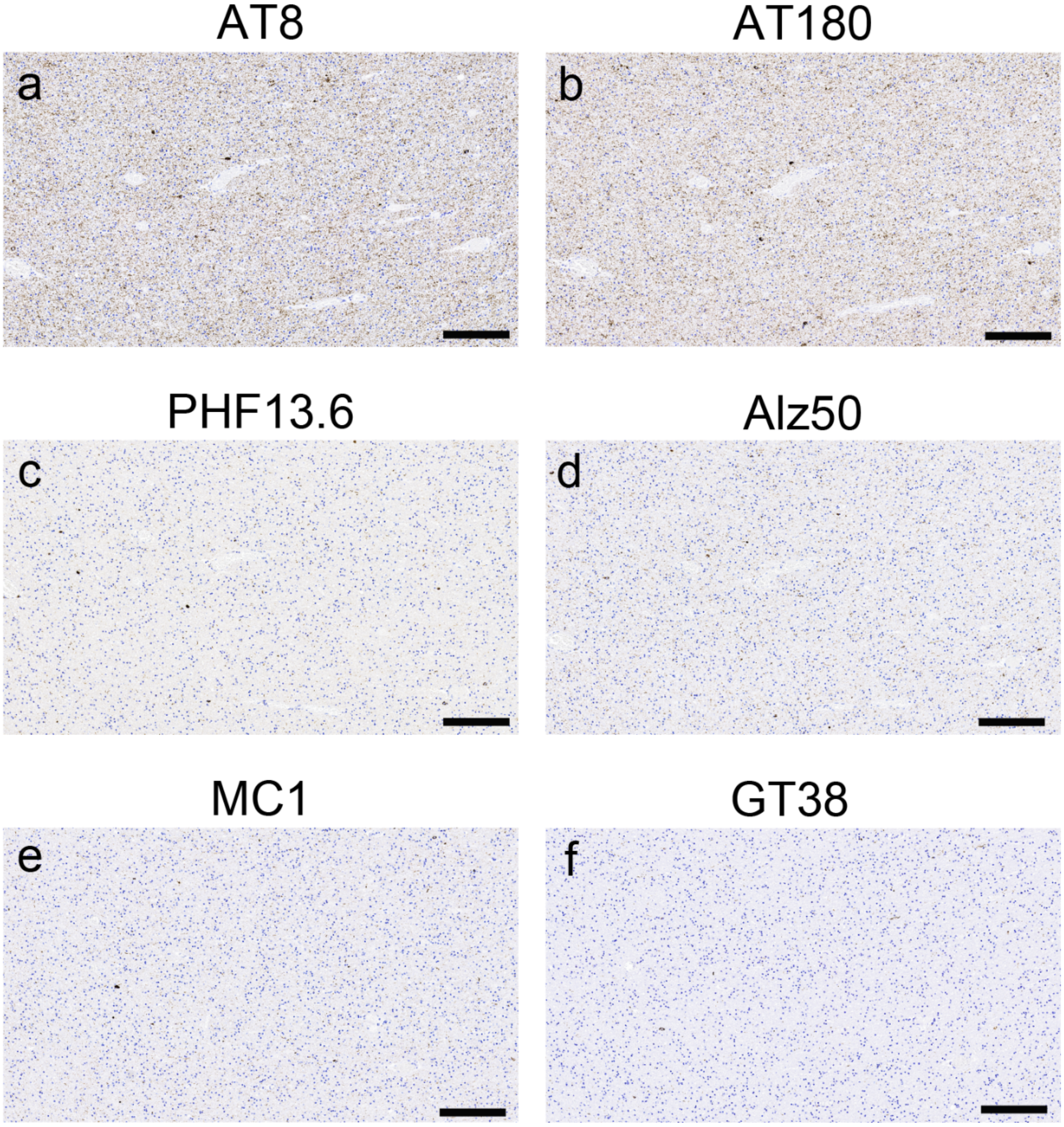

(a–f) Same areas in DS1.

Scale bar = 200 μm (a–f)

Supplementary Figure S3. Representative microphotographs and a summary of the results of tau seeding assay analysis

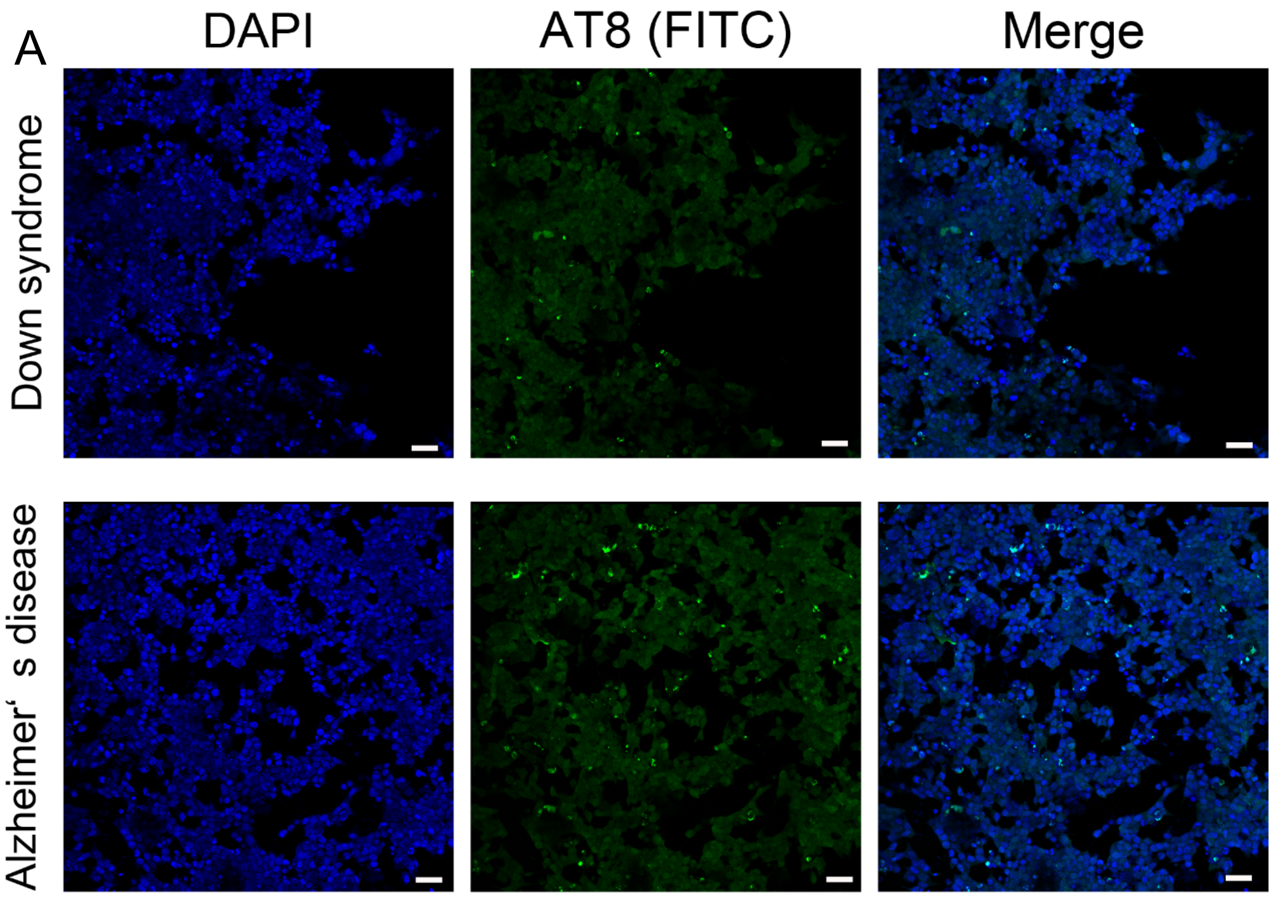

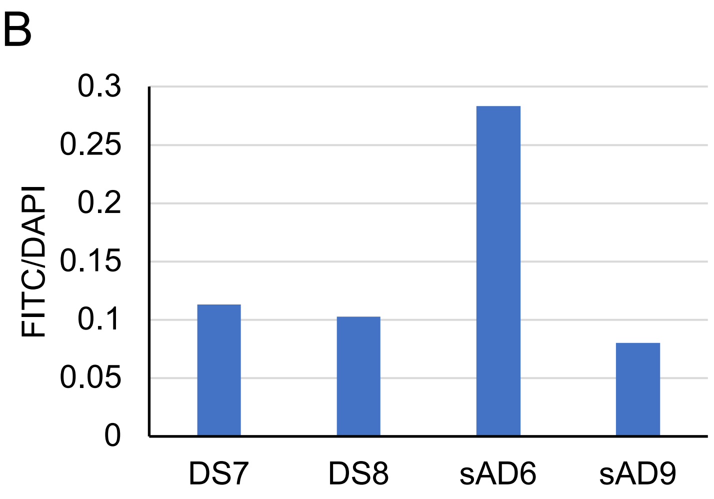

	FITC Count	DAPI Count	FITC/ DAPI	Ave.
DS7	380	1940	0.196	0.176
DS8	160	1058	0.151	
AD6	972	2307	0.421	0.278
AD9	341	2542	0.134	

(A) Confocal images. (B) Summary of the results.

In the temporal cortex, both Down syndrome (DS) and sporadic Alzheimer's disease (sAD) cases show the seeding activity consistent with the Braak NFT stage (DS7/DS8, Braak NFT stage 6/5; sAD6/sAD9, Braak NFT stage 6/4). Scale bar = 50 μm (A)

Supplementary Table S1. All results of quantitative analysis for Aβ

Case	Cerebrum (temporal cortex)						Cerebellum (molecular layer)					
	6F/3D		$A\beta_{42}$		$A\beta_{Np3E}$		6F/3D		$A\beta_{42}$		$A\beta_{Np3E}$	
	Ave-Size	P-Load	Ave-Size	P-Load	Ave-Size	P-Load	Ave-Size	P-Load	Ave-Size	P-Load	Ave-Size	P-Load
DS1	77.2	9.6	65.4	28.0	119.2	10.7	76.2	3.4	605.7	55.6	155.9	7.4
DS2	77.3	16.2	99.0	24.7	173.2	15.4	99.2	3.4	419.0	52.1	100.5	4.2
DS3	63.1	12.1	71.0	23.1	91.3	11.3	168.9	5.1	451.4	38.9	217.7	4.4
DS4	46.1	1.8	106.3	11.7	46.1	1.8	43.4	0.2	297.7	20.7	132.9	2.0
DS5	44.5	4.9	38.4	10.7	56.8	5.2	84.4	2.3	535.1	44.9	178.9	8.2
DS6	59.3	6.9	88.8	19.9	92.8	8.2	80.7	0.7	389.4	33.6	145.8	4.1
DS7	71.4	12.3	111.4	26.6	145.8	18.2	198.2	3.4	403.9	33.4	167.7	2.1
DS8	67.6	1.9	100.4	10.2	84.0	4.2	35.2	0.1	491.3	27.7	221.3	5.1
DS9	42.0	5.5	83.0	17.1	58.1	6.6	86.4	1.6	314.1	21.4	218.9	8.0
DS10	45.2	4.4	67.9	11.2	107.9	7.0	46.8	0.2	351.0	24.6	134.1	3.3
AD1	45.0	8.2	56.5	12.0	56.5	8.9	90.1	1.6	477.8	28.4	180.6	5.5
AD2	52.1	5.7	75.5	12.0	84.0	6.9	41.1	0.1	217.3	2.7	60.3	0.1
AD3	47.2	4.8	60.0	7.9	67.0	6.0	119.2	0.4	253.2	1.6	199.1	0.8
AD4	41.2	4.2	65.3	9.4	61.2	5.3	82.5	0.5	505.6	5.7	315.8	2.4
AD5	35.8	6.6	47.0	10.5	35.7	6.6	N/E	N/E	N/E	N/E	N/E	N/E
AD6	27.0	5.3	28.2	5.0	45.3	10.1	N/E	N/E	N/E	N/E	N/E	N/E
AD7	25.0	4.3	28.6	6.9	25.5	3.7	62.3	0.1	400.7	9.5	42.9	0.2
AD8	24.0	2.1	43.5	8.3	40.6	4.6	N/E	N/E	N/E	N/E	N/E	N/E
AD9	55.0	3.2	71.6	4.5	124.8	4.0	167.0	3.4	252.0	7.0	216.3	5.1
AD10	15.6	2.6	29.2	6.1	30.6	5.2	48.1	0.1	368.2	6.3	137.7	1.1

Abbreviations: AD, Alzheimer's disease; Ave, average; DS, Down syndrome; *N/E, not evaluated; P, plaque

* N/E means that we did not evaluate the Aβ burden in this case because the case did not show 6F/3D or Aβ42 or AβNp3E deposition in the molecular layer.

Supplementary Table S2. All results of quantitative analysis for tau

Case	AT8		AT180		PHF13.6		Alz50		MC1		GT38	
	Cx	WM	Cx	WM	Cx	WM*	Cx	WM	Cx	WM	Cx	WM*
DS1	76.7	10.9	70.7	10.1	45.6	1.9	12.6	2.6	30.6	1.6	8.6	0.3
DS2	48.0	8.0	38.3	8.9	14.5	3.6	3.8	1.2	1.3	0.7	0.1	0.0
DS3	74.1	5.8	70.0	4.8	43.2	1.4	6.2	1.6	6.4	0.6	6.3	0.1
DS4	16.9	1.0	12.4	0.9	1.0	7.3	1.3	0.3	0.4	0.2	0.0	0.1
DS5	44.0	3.6	58.9	5.0	20.6	5.3	3.2	0.9	1.0	0.3	5.3	0.1
DS6	74.9	4.1	73.9	4.1	24.5	6.5	5.8	0.9	4.6	0.2	0.9	0.2
DS7	57.9	6.8	53.5	6.6	2.9	11.8	2.8	1.0	2.1	0.4	0.2	0.1
DS8	37.9	5.0	32.5	4.4	0.9	14.5	2.0	0.8	0.9	0.6	0.1	0.9
DS9	77.0	5.5	66.9	5.0	36.6	2.6	9.1	0.9	8.4	0.2	6.3	0.1
DS10	61.3	9.5	56.6	8.4	26.8	13.1	10.1	1.6	7.1	0.6	3.5	0.1
AD1	52.5	3.4	45.9	2.8	21.4	1.4	4.8	1.2	1.4	0.6	0.6	0.3
AD2	52.9	2.2	46.9	2.8	24.2	5.5	3.3	0.4	6.3	0.5	0.7	0.7
AD3	43.7	4.6	36.5	4.0	19.4	2.4	3.3	0.9	2.9	0.8	0.1	0.0
AD4	47.9	3.0	37.5	2.7	11.2	9.5	1.9	0.5	0.6	0.3	0.4	0.2
AD5	65.2	2.9	56.2	3.2	38.7	1.1	6.2	0.8	2.2	0.2	2.4	0.2
AD6	32.7	2.6	22.7	1.8	12.5	0.6	6.0	0.8	1.8	0.3	0.6	0.0
AD7	58.5	1.0	52.8	0.9	20.6	0.2	7.3	0.4	4.3	0.2	2.2	0.0
AD8	47.1	1.5	42.9	1.4	21.7	1.6	1.1	0.5	0.2	0.2	0.9	0.2
AD9	15.3	0.5	13.7	0.6	0.2	9.7	1.0	0.1	0.4	0.2	0.0	3.7
AD10†	42.3	0.6	46.2	1.1	3.3	9.2	2.2	0.1	0.3	0.1	2.2	0.3

Abbreviations: AD, Alzheimer's disease; Cx, cortex; DS, Down syndrome; WM, white matter

* Data shown in grey are abnormally high compared to the bright-field observation, which may be due to nonspecific immunoreactivity in the background. These data were excluded from the statistical analysis in this study (GT38). Regarding the results of PHF13.6 in the WM, we did not statistically evaluate them because half of the results were abnormally high.

† The results of the tau deposition burden obtained from AD10 were excluded from the statistical analysis of tau pathology.
